# Teachers’ online preparedness in times of crises: trends from Norway and US

**DOI:** 10.1007/s10639-023-11733-5

**Published:** 2023-04-11

**Authors:** Dawn M. Hathaway, Greta B. Gudmundsdottir, Matthew Korona

**Affiliations:** 1 Division of Learning Technologies, George Mason University, Fairfax, VA USA; 2 Department of Teacher Education and School Research, University of Oslo, Oslo, Norway

**Keywords:** Digital competence, Teacher education, K-12 schools, Online teaching, Nordic context, US context

## Abstract

The closing of schools world-wide in March 2020 due to the COVID-19 pandemic resulted in a rapid and unexpected shift from predominantly in-person teaching to online teaching practices. As teacher educators in the field of educational technology, we wondered about the preparedness of teachers for making the transition to fully online environments. Through an internationally distributed survey consisting of predominantly open-ended questions, we captured teachers’ perceptions of this transition. We aimed to inform our practice and that of other teacher educators about the strengths and weaknesses of professional development designed to develop teachers’ digital competence. In this paper, we present data from Norwegian (n = 574) and US (n = 239) teachers related to their elaborations on readiness. We qualitatively examined data for evidence of extent of preparedness and alignment to the pedagogical, ethical, attitudinal, and technical dimensions of digital competence. Findings indicated themes related to extent of preparedness, trends in preparation, focus on digital tools, teacher agency without autonomy, collaboration/networks, and challenges for work and learning lives. Findings informed implications and recommendations for the professional development of teachers’ digital competence at the teacher education, K-12 schools, and school policy/leadership levels.

## Introduction

The closing of schools world-wide in March 2020 due to the COVID-19 pandemic resulted in a rapid and unexpected shift from predominantly in-person teaching to online teaching. While online teaching applies some of the similar knowledge and skills as in-person teaching (Lee & Hirumi, 2004), extensive literature points to the need for additional and special online teaching knowledge and skills (e.g., Baran et al., 2011; Darabi et al., 2006; Goodyear et al., 2001; Rehn et al., 2018). Online teaching knowledge and skills include both pedagogical and technical conceptualizations that impact all aspects of the online learning environment such as content, activities, organization, interactions, communications, assessment, and group work (Gloria & Uttal, 2020; Lee & Hirumi, 2004). Additionally, a shift from an in-person setting to an online setting requires teachers “to adapt to new roles for creating effective and meaningful learning experiences” (Baran et al., 2011, p. 425). Hence the digital competence of teachers reflects the ability to teach across multiple modalities.

As teacher educators in the field of educational technology, we wondered about the readiness of teachers for making the rapid transition to fully online environments at the onset of the pandemic and in particular, their sense of preparedness. We initiated the Teachers’ Readiness Online (TRIO) project (Gudmundsdottir & Hathaway, 2020a) through an internationally distributed survey to capture teachers’ perceptions of their preparedness to teach online and strategies to include vulnerable learners in their online teaching practices. We aimed to inform our practice and that of other teacher educators about the strengths and weaknesses of professional development designed to develop teachers’ digital competence.

A number of studies have focused on teachers’ readiness and preparedness to teach online amid the pandemic. The focus of these studies include quantitative studies in higher education (e.g., Scherer et al., 2020), quantitative studies in secondary education (e.g., Howard, et al., 2021), qualitative studies in select secondary education disciplines (e.g., Sengıl Akar, & Kurtoglu Erden, 2021), and several studies that were country/state- specific (e.g., Eadens et al., 2022, Fatimawati & Badiozaman, 2021; Li, 2022). Readiness was also studied through the lens of TPACK (e.g., Li, 2022; Scherer, et al., 2020). Eadens et al. (2022) examined US teachers’ perceptions of readiness, primarily through quantitative methods to determine differences in teacher perceptions of preparedness among teacher demographics as well as among levels of support from universities, districts, and schools. In addition, five open-ended questions structured responses focused on challenges and successes during the shift to online teaching as well as suggestions on how schools/districts/universities could have prepared teachers differently or more effectively.

The present study is unique in that it focused on primary and secondary (K12) teachers and a qualitative approach to capture teachers’ voices on their perception of preparedness to teach online at the onset of the pandemic. Although we collected perspectives from 1186 teachers from 36 countries about their experiences related to online teaching in the early weeks of COVID-19 school closures, the majority of participants were from Norway (n = 574) and the US (n = 239). Therefore, our current work focuses on examining data from these two countries. Furthermore, the pre-pandemic focus in Norway on digital competence overall and focus in the US specifically on developing online teachers as indicated in the literature that follows, presents an interesting study of preparedness. Finally, as teacher educators in Norway and the US, we were curious to know about teachers’ online readiness and digital competence in a more global context to inform our practice and that of other teacher educators. Are teacher educators on different continents faced with similar issues? Are there common or different potentials that we might share or learn from each other? This study addresses the emergent issues regarding teachers’ digital competence and professional development. Findings from a preliminary study highlighted that despite Norwegian and US teachers’ inexperience and unpreparedness for conducting their teaching practice solely online, they were prepared to use various digital tools and willing to make online learning work for them and their students (Gudmundsdottir & Hathaway, 2020b). In the present study, we extend our examination of Norwegian and US teachers’ elaborations on preparedness for teaching online through the lens of digital competence to uncover common themes and nuances among and between Norwegian and US data. The research questions that guide this study were:

1. What did Norwegian and US teachers report about their preparedness to teach online at the onset of school closings?
2. How did Norwegian and US teachers’ elaborations on their preparedness to teach online at the onset of school closings align with pedagogical, ethical, attitudinal, and technological aspects of digital competence?

## Pre-pandemic literature on Digital competence and K12 online teaching

The purpose of this section is to highlight the pre-pandemic literature related to developing teachers’ digital competence and more recently in the last decade, supporting digital competence that includes K12 online teaching. This literature has informed teacher education in Norway and the US. This body of work provides insights as to expectations in terms of readiness and preparedness to teach online and opens the door for potential commentary on alignments between pre-pandemic preparation and preparedness at the onset of the pandemic. For this reason, we have intentionally omitted research published during the pandemic as emergency remote teaching may have impacted specific constructs that interfere with notions of preparedness at the onset of the pandemic (Tawfik et al., 2021).

### Digital competence

The variety of different concepts to identify and describe students’ and educators’ proficiency when using digital technology for teaching and learning purposes is vast. These are concepts such as *digital skills* (Tsekeris, 2019; Pérez-Escoda et al., 2016; Goldhammer et al., 2013); *ICT skills* (Kaarakainen et al., 2017), *Digital literacy* (Buckingham, 2015; List, 2019; Eshet-Alkalai, 2004), *digital pedagogy* (Kivunja, 2013; Stommel, Friend & Morris 2020) and *digital competence* ( Gudmundsdottir & Hatlevik, 2018; Krumsvik, 2014; Petterson 2018). The different concepts are often a combination of a domain part (i.e., digital, ICT) and a knowledge perspective (i.e., skills, literacy, competence) (Hatlevik et al., 2015) but in terms of content of the concepts, they are often overlapping and used in a similar manner. Digital competence is a concept that is widely used in Europe and in particular in the Nordic countries where *professional digital competence* is accorded much importance (Brevik et al., 2019; Gudmundsdottir & Hatlevik, 2018;  Lund et al., 2014).

The European Commissions’ DigComp framework was initiated for developing and understanding digital competence in Europe (Ferrari, 2013) and has been widely used as a reference for policy makers in Europe. In 2016 the framework was revised and a DigComp 2.0 version was available with further development of the conceptual reference framework, now including eight proficiency areas (Vuorikari et al., 2016) and the DigCompEdu framework (Redecker, 2017). Furthermore, the Digital Education Action Plan (2021–2027) outlines the visions of the European Commission for digital education across Europe. The plan involves six target areas divided into 22 distinctive competences (European Commission, 2021) Policy frameworks highlight the political will, aims to be achieved and the potential of the technology but are less entrenched in research. Consequently there are various ways to define a concept such as digital competence. All highlight different aspects of various frameworks (Ilomäki et al., 2011) as well as different approaches and stakeholder views. Olofsson et al. (2020) noticed that a major theme within previous research is defining digital competence and Ilomäki et al. (2016) described digital competence as a “boundary concept” (p. 670) in educational policy and practice but also between different disciplines. One thing is clear though; there has been a shift from skill based definitions considering digital skills as a simple proficiency that can be trained to a more complex competence based emphasis. Where competence is “…more than just knowledge and skills. It involves the ability to meet complex demands, by drawing on and mobilising psychosocial resources (including skills and attitudes) in a particular context” (OECD, 2005, p. 4).

**Fig. 1:**
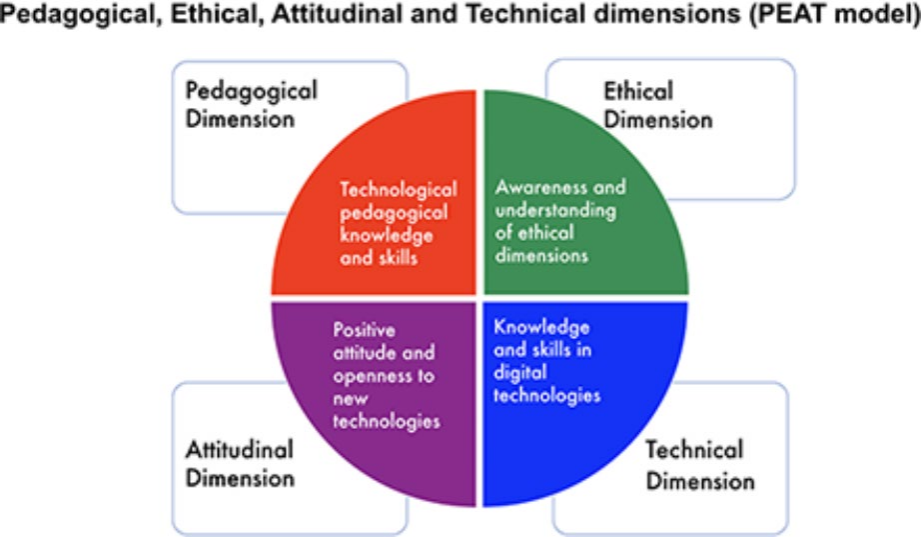
The four dimensions of the PEAT model for digital competence (DICTE, 2019)

McGarr and McDonagh (2019) conducted an extensive literature review of digital competence models and frameworks used internationally and identified their strengths and weaknesses. Findings showed a limited focus on ethical and attitudinal aspects of digital competence. In addition, most models were found to have a hierarchical way of viewing digital competence with levels, key criteria, an advancement, or progression of some sort. The PEAT model (DICTE, 2019) originated from an Erasmus + project where the aim was to develop an alternative digital competence framework which included elements that the project partners (including the second author) had identified as necessary for teachers and teacher educators. The model was developed as a synthesis of existing models to capture the main dimensions in teachers professional digital competence (McGarr & McDonagh, 2019) but also allowed for dimensions missing in existing frameworks. The PEAT model conceptualizes key dimensions of digital competence for teachers and teacher educators through four equally important and interconnected dimensions. The *pedagogical* dimension relates to pedagogical design and practices with technology use in different subjects and professional practice. The *ethical* dimension focuses on issues such as online responsibility, privacy, critical use of resources, plagiarism, and copyright issues. The *attitudinal* dimension attends to teachers’ attitudes toward technology, their ability to adapt technology use in their practice as well as agentic stance when using digital technology. The *technical* dimension refers to the ability to use software and hardware and understanding how technological devices operate. An illustration of the PEAT model is provided in Fig. 1.

### Teaching Digital competence Guidelines/Standards

For decades, school districts, governments, and other stakeholders around the globe have invested in digital technologies for schools (Kearney et al., 2018). In the US, the use of digital technologies in schools became a national priority in 1983 as part of the solution for educational reform (Bakir, 2016). School-based, technology driven initiatives and tools have focused on access, delivery, and pedagogy (e.g., 1:1 and laptop computing, online and blended learning, learning management systems). Similarly, the use of digital technology in Norway is well established in schools through 1:1 initiatives, necessitating attention to digital skills and knowledge. Already in 2006, the Knowledge Promotion Reform laid the foundation for five basic skills, namely reading, writing, numeracy, oral (dissemination) skills and digital skills (Directorate of Education and Training, 2012). These basic skills are seen as fundamental for both school, work and leisure time and should prepare for students’ learning in all subjects and in grades 1–13 (primary – secondary school). Digital skills are thus perceived as the competences necessary for students’ learning in school, for their future work and social life. The Directorate of Education and Training consequently developed the framework for basic skills which describes students’ five competency areas (where digital skills are also organized in progressing five levels. This framework has been important for teachers in Norway when considering which aspects and what emphasis to do when implementing digital technology in their teaching.

As for teacher standards the Directorate of Education and Training established a professional digital competence framework for teachers (PDC) in 2017. The framework was intended to further support how teachers can work with students’ digital competence. The framework describes various areas of knowledge and skills, which are part of teachers’ professional digital competence (PDC) (Kelentrić et al., 2017). The framework categorizes by knowledge, skills and competence aspects related to seven main areas: (1) subjects and basic skills; (2) the school in the community; (3) ethics; (4) pedagogy and subject didactics; (5) management of learning processes; (6) interaction and communication; (7) change and development. After years of using digital technology and developing students’ digital competence in the classroom researchers pointed at the necessity to establish a common set of terminology to be used by teachers, student teachers and teacher educators. The framework thus provides a policy view and understanding of what the term PDC means (Kelentrić et al., 2017). The PDC framework for teachers is first and foremost a policy document describing political will and emphasis on digital competence rather than a research based framework building on empirical evidence. Several researchers have also pointed at the need for more emphasis on ethical and attitudinal aspects (Gudmundsdottir & Hathaway, 2020b; McDonagh et al., 2021) as well as the importance of transformative agency or transformative digital competence (Brevik et al., 2019; Lund et al., 2019).

In the US, all 50 states have adopted at least one set of standards developed by the International Society for Technology in Education (ISTE) (International Society for Technology in Education, 2020). In 2017, the ISTE Standards shifted from focusing on digital tool competence to effectively integrating digital tools to provide instruction (Trust, 2017). The ISTE Standards are placed into categories specifically for students, educators, educational leaders, and coaches. Particularly relevant to the present study are the standards for students, educators, and education leaders. ISTE Standards for Students (International Society for Technology in Education, 2016) aim to equip students with skills to effectively participate in digital environments and include empowered learner, digital citizen, knowledge constructor, innovative designer, computational thinker, creative communicator, and global collaborator. Technology competencies for teachers are embedded in the ISTE Standards for Educators (Carpenter et al., 2020; International Society for Technology in Education, 2017). These standards focus on the necessary digital competency and pedagogy to effectively integrate technology. The ISTE Standards for Educators identify seven educator qualities and attributes essential to developing empowered learners: learner, leader, citizen, collaborator, designer, facilitator, and analyst. ISTE Standards for Education Leaders (International Society for Technology in Education, 2018) focus on overseeing the district- or school-wide implementation of digital learning and include equity and citizenship advocate, visionary planner, empowering leader, systems designer, and connected learner.

The ISTE Standards have informed the teacher preparation programs (Parra et al., 2019) and courses intended to increase digital competence as well as served as a framework for exploring preservice teachers’ confidence in technology competence. Research focused on preservice teachers’ confidence in their technology competency showed low confidence in leaders, designer, facilitator and analyst and higher confidence among those who had some teaching and team-teaching experience (Kimm et al., 2020).Thus, the ISTE Standards offer a starting point to prepare teachers for technology integration and measure their preparedness.

To meet the needs of future-ready learners, it is no longer enough for teachers to be prepared for technology integration. Teachers need to be prepared to teach online (Cooper et al., 2020). Many teachers already have the prerequisites for teaching online noted in the literature: subject-matter expertise, experience with in-person teaching, and experience as an online learner (Archambault & Crippen, 2009; Hathaway & Norton, 2012). The intention to introduce online teaching competency and standards in this study is not to claim that teaching online during the pandemic was the same as traditional online education or online teaching and learning (Hodges et al., 2020). Rather, we treat online teaching competency as a possible path toward teachers’ professional digital competence. For that reason, we situate readiness for teaching online in that literature with hope of gaining insights in previous ways of teaching online.

The inclusion of online and blended learning strategies for primary and secondary education students in both Norway and the US had been growing steadily prior to the pandemic (Allen & Seaman, 2014; Directorate for Higher Education and Skills, 2022; Johnson et al., 2014; Tønnesen, 2022; Yu & Hu, 2016). To promote effective online teaching and learning, the International Association for K-12 Online Learning (iNACOL) presented national standards for quality online teaching as early as 2008 (International Association for K-12 Online Learning, 2011). By 2019, Quality Matters and Virtual Learning Leadership Alliance (2019) had enlisted experts from the field of K12 online learning to refresh, revise, and update the standards. These standards centered on eight categories representing professional responsibilities, digital pedagogy, community building, learner engagement, digital citizenship, diverse instruction, assessment/measurement, and instructional design. Blended teaching, which bridges online teaching with in-person teaching, is also informed by a set of competencies. The Blended Learning Teacher Competency Framework describe characteristics of competent blended teachers organized under four domains: Mindsets (vision, orientation toward change), Qualities (grit, flexibility, transparency, collaboration), Adaptive Skills (reflection, continuous improvement, innovation, communication), and Technical Skills (knowledge of data practices, instructional strategies, management of experiences, instructional tools) (Powell et al., 2014, p. 8).

Calls for preparing teachers to teach in online and blended environments began to emerge in the last decade (e.g., Baran, et al., 2011; Barbour et al., 2013) with an emphasis on the development of specific knowledge and skills required for teaching online (Moore-Adams et al., 2016). Despite these calls, few programs are available that specifically prepare K12 teachers for teaching online (Shephard et al., 2016). A majority of undergraduate teacher preparation programs do not formally prepare teacher candidates to teach online due to an already overcrowded curriculum (Graziano & Bryans-Bongey, 2018) or because traditionally online teaching is targeting higher or adult education rather than K-12. Additionally, there is a lack of competency among teacher educators to model the use of online and blended teaching (Graziano & Bryans-Bongey, 2018).

### Barriers

To better understand teachers’ preparedness to teach online at the onset of the pandemic, it is important to acknowledge the barriers to effective digital technology use identified in the decades prior to the pandemic. Early on, Ertmer (1999) had described first-order barriers as extrinsic including lack of access to digital tools (hardware and software), not enough time allotted due to other instructional responsibilities for the reflection and planning needed to implement digital tools into instruction and limited to no support from administrators or technology staff. First-order barriers are easy to identify and remedy through acquisition of digital tools or implementation of teacher learning experiences. Conversely, second-order barriers are intrinsic to teachers such as their pedagogical beliefs and practices, disposition toward technology integration, and inclination for trying new instructional approaches.

Second-order barriers are more difficult to overcome than first-order because teachers often have pre-established, deep beliefs about their pedagogy. Teachers may weigh first-order barriers differently. For example, teachers may not have sufficient training yet believe they are capable of integrating technology in their practice, or they might wait to integrate technology until more training is acquired. As another example illuminated by Ertmer (1999), teachers may slowly implement technology into instruction due to lack of knowledge, or they might task students with teaching classmates the functionality of classroom technology. While first-order barriers have lessened (Durff & Carter, 2019) with the influx of digital tools, reliable internet access, and sufficient bandwidth in schools such as those in Norway and the US, second-order barriers persist (Tondeur et al., 2017).

Park and Ertmer (2007) suggested that collaboration with peers could alleviate barriers. Bai and Ertmer (2008) determined that taking an introductory educational technology course was helpful in improving preservice teachers’ technology attitudes related to educational benefits. Tsai and Chai (2012) introduced a third-order barrier, a lack of design thinking. While lack of access and teacher beliefs/attitudes are both barriers to technology integration, a lack of design thinking can hinder teacher ability to integrate technology into instruction. Access to digital tools and instructional support for implementation as well as willingness to integrate technology to enhance instruction does not necessarily guarantee educationally rich classroom experiences. Thus, teachers must leverage digital tools to “re-organize or create learning materials and activities, adapting to the instructional needs for different contexts or varying groups of learners” (Tsai & Chai, 2012, p. 1058).

Muilenburg and Berg (2003) indicated the top 12 strongest barriers to distance education as ranked by K12 educators. Listed in order from greatest to least were increased *time commitment*, *lack of money to implement programs*, *organizational resistance to change*, *lack of shared vision for distance education in organization*, *lack of strategic planning for distance education*, *lack of training provided*, *lack of technology enhanced classrooms*, *slow pace of implementation*, *lack of grants*, *lack of technical support*, *difficult to convince stakeholders of benefits*, and *lack of support staff to help course development*. From the 64 barriers identified by Muilenburg and Berg, K12 educators ranked *lack of personal technological expertise* and *ethical issues* were ranked by at the bottom of the list (#59 and #64, respectively), denoting these as weaker barriers. Barriers to K12 online teaching have recently been investigated but more recently (e.g., Jimoyiannis & Koukis, 2023), but this more recent work may have been impacted by specific constructs that interfere with notions of preparedness at the onset of the pandemic (Tawfik et al., 2021). In this paper, we examined barriers that teachers identified to be present *at the onset* of emergency remote teaching to better understand teachers’ preparedness, how previously identified barriers were at play and to inform a path forward.

## Methods

In this section, we discuss the methodological approach of this paper and highlight the most important issues related to our data analysis. This study was submitted to the George Mason University Institutional Review Board (IRB) Office and determined exempt from IRB review. In addition, The Norwegian Centre for Data Services were conferred with but as the online survey is without any personal data, the study was exempt from a formal registration process. The TRIO survey (Gudmundsdottir & Hathaway, 2020a) was a researcher-created, eight-item questionnaire that consisted of both closed and open-ended questions. Four closed-ended questions addressed consent, frequency of online teaching, country, and teaching level. Four open-ended questions that addressed elaboration on preparation, measures by schools/teachers to include vulnerable learners, additional comments on challenges and/or opportunities. Regarding content validity, relevance, and reliability of the survey tool, the questions on the survey were derived from those posed by the Norwegian Directorate (personal communication, March 20, 2020). Additionally, we had the questions reviewed by a content expert from each country. Open-ended questions were used so as not to limit the possible answers that teachers could provide. In other words, teachers provided their own perspectives rather than be limited by the assumptions of the researchers.

The TRIO survey was translated from English into Norwegian, German, French, Italian, Spanish, Estonian, Icelandic, Romanian and Chinese with assistance from native speaking colleagues. It was distributed through the authors’ social media sites (e.g., Facebook) and listservs (e.g., professional organizations) and opened from March 31, 2020 to April 28, 2020.

Through the TRIO survey, we collected perspectives from 1186 teachers from 36 countries about their experiences related to online teaching in the early weeks of COVID-19 school closures. Because the majority of participants were primary and secondary teachers from Norway (n = 574) and the US (n = 239), our current work focuses on examining data from these two countries. The data sources for this study were responses from two open-ended questions that asked teachers to (1) elaborate on how prepared they were to teach online and (2) add anything else relevant to understanding their teaching practice during this crisis (for example challenges and/or opportunities you and/or your colleagues have encountered with the digital, lesson planning, school hours, how to follow-up learners, learners’ attendance, handing in assignments, various routines additional comments on challenges and/or opportunities). Responses from the Norwegian language survey were translated into English by the second author who is fluent in Norwegian and English.

### Data Analysis

We selected a qualitative approach to analyze the responses to the open-ended questions related to elaborations on preparation for online teaching and comments on challenges and/or opportunities. Qualitative analysis procedures emphasize the views of the participants and interpret the subject of study from their perspective. This process is inductive in that themes are generated during the process of categorizing, coding, and organizing data (Maxwell, 2013).

First, teachers’ elaborations on the extent to which they felt prepared to teach online were emically coded using the most prevalent terms found in the data sets serving as subsets of the theme Extent of Preparedness: *Very Well-Prepared*, *Prepared*, *Not Prepared*, *No Indication*.

Second, we confirmed and assigned a preparedness term to each teacher based on our coding of the elaboration statements. While emically coded, the extent of preparation designations were developed from our interpretation of teachers’ open-ended responses. In nearly all cases, teachers’ elaboration comments explicitly stated the extent of their preparedness (e.g., “I am quite well prepared). In a very few cases (e.g., I’m becoming more and more prepared as I have sat through tons of trainings,” coded as *unprepared*), designations were confirmed as a result of triangulation. We returned to the full text of the original elaboration as well as the response to the open-ended question soliciting additional information to find further evidence for a designation (e.g., the designation, *unprepared*, was supported by the additional comment, “not prepared for certain tasks).

Third, we quantitized the total number of codes within each Preparedness subtheme and calculated percentages to highlight similarities and differences between the Norwegian and US data sets with regard to teachers’ reported sense of preparedness to teach online. (Saldaña, 2016).

To analyze how Norwegian and US teachers’ elaborations on their preparedness to teach online at the onset of school closings aligned with pedagogical, ethical, attitudinal, and technological aspects of digital competence, we used the PEAT model (DICTE, 2019) as an analytical framework. The strength of the PEAT model as an analytical framework is that it provides a straightforward and applicable framework when viewing the interconnectedness of the four dimensions of teachers’ digital competence. Whereas other competency models are rather specific and detailed, the PEAT in its simplicity presents the absence of specificity and instead “allows for autonomy and agency and for local interpretations of the four dimensions to be enacted” (McDonagh et al., 2021, p.14). Also, the emphasis on the ethical and the attitudinal dimensions in developing teachers digital competence in addition to the more traditional focus on technical skills and the pedagogical use of digital technology is one of the strengths of the PEAT model.

For the categorizing and coding process, we independently examined survey responses to the two open-ended questions that asked teachers to (1) elaborate on how prepared they were to teach online and (2) add anything else relevant to understanding their teaching practice during this crisis. The Norway and US data sets were examined separately. Using the PEAT (DICTE, 2019) dimensions as pre-established categories to identify connections to aspects of digital competence, we independently coded teachers’ elaborations on their preparedness for online teaching. We fractured coded statements by entering them with associated codes into a spreadsheet. Statements included on the spreadsheets were then organized under a PEAT category. For example, a coded statement, “Privacy has ironically made it extremely difficult” was placed under the Ethical category as the code aligned with language used to define the ethical dimension of PEAT. Table 1 presents a summary of the PEAT pre-established category definitions used to align and categorize codes.

**Table 1:** Summary of PEAT pre-established category definitions

Pedagogical	Ethical	Attitudinal	Technological
pedagogical design and practices with technology use in different subjects and professional practice	online responsibility, privacy, critical use of resources, plagiarism, and copyright issues	attitudes toward technology, ability to adapt technology use in practice as well as agentic stance when using digital technology	ability to use software and hardware and understanding how technological devices operate.

We next compared our coded statements with each other to ensure the accuracy of the coding process and establish consensus coding. When a statement was identified by only one of us, we returned to the statement in context, and agreed to either include the statement or to eliminate it. We then compared the categories to which we had assigned statements. When our coding of statements differed, we returned to the elaboration statement, examined the statement in context, and agreed upon an appropriate category.

After coding and organizing teachers’ elaborations on readiness into PEAT pre-established categories, we used thematic coding to identify mutual themes across the two data sets. We did this dynamically and collaboratively. The main themes generated were *Trends in Preparation*, *All about Tools*, *Agency without Autonomy*, *Influence of Collaboration and Networks*, and *Challenges in Work/School Life.* For example, upon examination of coded statements under the technological PEAT category, “a crash course on online teaching tools” informed the theme, *Trends in Preparation*. A summary was developed for each theme to emphasize the connection between the theme and digital competence as defined by the PEAT model. These summaries are presented in the Findings section.

Finally, we returned to the statements in context and collaboratively selected quotations for each theme to reflect teachers’ voices. Selection was based on consensus that a quotation reflected strong patterns in the data, represented alignment to PEAT dimensions, was distributed across Norwegian and US teachers to fully represent the data sets, and distributed across teachers’ reported level of preparedness. Selected quotations represented teachers’ voice in the findings while contributing to data credibility and transparency of the research.

### Limitations

In order to maintain anonymity and address ethical concerns, a comparative study of the perceptions of Norwegian teachers and US teachers was not possible on an individual level. However a comparison of the readiness and reflections from teachers on a country level is made.

One of the limitations may be that teachers’ perspectives were collected during the early weeks of the Covid-19 pandemic in chaotic times. The data we have are therefore not showing the development in their digital competence during the pandemic and the home-schooling periods. Rather, it only shows their reflections on their readiness during April 2020.

Also, this study, which asks teachers to elaborate on how prepared they were to teach online, relies on self-reported data. Self-reported data can make it difficult to distinguish between teachers’ perceptions and their actual behaviors. We tried to account for this through careful word choice on the survey and offering two opportunities for responses to open-ended questions. In the majority of cases, asking teachers to *elaborate* resulted in an example of behavior, a definition of what they meant, or a story to justify their statements regarding preparedness.

Finally, we acknowledge the possibility that similar concepts and terminology related to digital competence are used in both countries but might carry slightly different meanings and definitions. One such example are the terms *competence* (Norway/European Union) and *competency* (US). We accounted for this by combining and presenting literature that informs work in each country and applying the terms as presented in the literature. In the process, we have greatly expanded our own thinking about teachers’ readiness for online teaching. With regard to data analysis, we tried to account for the potential terminology differences between the countries through conversations about terminology differences during the coding process as well as using a framework that we have used together in prior collaborations and research.

## Findings

### Extent of preparedness

When asked how prepared they were to teach online at the onset of school closings, more than half of Norwegian teachers (59.1%) and US teachers (52.9%) indicated they were prepared. We collapsed the very well-prepared and prepared codes to obtain an overall percentage of preparedness as very well-prepared only describe a degree of being prepared. We also wanted to showcase that there were teachers who specifically stated they were very well-prepared.

A larger percentage of Norwegian teachers (11.0%) than US teachers (2.0%) specifically stated they were well-prepared to teach online. The descriptions found in the elaborations of Norwegian teachers to support being *very well-prepared* included references to “having digital competence,” access to digital technologies, routine use of technology-based strategies in the classroom and/or across the school prior to the pandemic, experiences an online learner, and formal coursework focused on digital competence. Descriptions in US teachers’ elaborations to support being *very well-prepared* included access to digital technologies, routine use of technology-based strategies in the classroom and/or across the school prior to the pandemic, creativity, and graduate degrees that focused on integration of technology in schools. Table 2 presents a summary of the sub themes generated from the overarching theme *Extent of Preparedness*.

**Table 2:** Percentage of Norwegian and US teachers’ extent of preparedness subthemes

Preparedness Subthemes	Norwegian teachers (N = 574)	Norway %	US teachers (N = 239)	US %
Very Well-Prepared	63	11.0%	4	2.0%
Prepared	276	48.0%	120	50.2%
Total Prepared	339	59.1%	124	52.9%
Not Prepared	207	36.0%	109	46.0%
No Indication	28	4.09%	6	2.5%

Throughout the next section teachers are described as *prepared*, *unprepared, or no indication*. *Prepared* includes well-prepared teachers unless otherwise noted. These italicized descriptors reflect instances where findings and/or representative quotations are aligned with teachers’ reported level of preparedness.

### Further insights from Elaborations

Analysis of teachers’ elaborations on their readiness to teach online through the lens of pedagogical, ethical, attitudinal, and technical dimensions of digital competence generated additional themes related to the extent of their preparedness. The themes provided further insights about influences on their sense of preparedness or unpreparedness.

#### Trends in Preparation

Among the teachers who indicated being prepared for the transition to online teaching, there were indications in elaboration statements as to how they came to be prepared. The theme, *Trends in Preparation* highlights the trends that influenced teachers’ perspectives of preparedness.

One trend referenced by teachers was structured teacher professional development at the district/municipality/school levels prior to and unrelated to the pandemic crisis. Teachers qualified their extent of preparedness with mentions of district/municipality/school-led training that supported technology-driven initiatives in place before the crisis.

“Every student in our district has an iPad to use at school and home. We have had training on using digital platforms… .” (*prepared* US)

“I am trained to use the students’ learning management platform (It’s Learning).” (*prepared* Norway)

There were many references through the elaboration statements across both countries to school-led emergency training that was offered at the onset of the pandemic or school closings:

“By ‘prepared,’ I am referring to a crash course in online teaching tools.” (*unprepared* US)

“Had basic training in our learning management platform. Publishing announcements, [how to] give feedback and send messages.” (*prepared* Norway)

Among US teachers identified as *unprepared*, there was evidence to support that school-led emergency training did influence a shift from feeling unprepared to an emerging sense of preparedness: “I’m becoming more and more prepared as I have sat through tons of trainings.” *(unprepared* US). Norwegian teachers who reported feeling unprepared at the onset of school closing, did indicate a shift towards preparedness, however, were not explicit that school-led emergency training facilitated that shift, “Little prepared in advance, but have learned a few things along the way about the use of Teams” (*unprepared* Norway). Given the numerous instances of Norwegian teachers referring to “Teams training” at the onset of the pandemic, it is likely that many learned to use “Teams” for teaching online through school-led training efforts. However, statements related to prior and crisis-driven training at the district/municipalities/school level were heavily focused on digital tool use. In contrast, education at the university level provided a foundation for not only using digital tools, but also the pedagogical considerations associated with digital learning.

Although not prevalent, a trend that emerged from this theme related to how teachers were prepared was the influence of university degrees and experiences. Teachers from both the US and Norway who connected a university experience to their preparedness and digital competence, also provided insights about their learning and knowledge gained:

“I received my Masters in [designing digital learning] at [University] last spring. That educational program prepared me from a theoretical and technical perspective to teach online to my students.” (*prepared* US)

“Through pedagogical training I learnt how to use digital resources” (*prepared* Norway)

“I am newly [sic] graduated high school teacher from [University], and we had a lot of focus on [professional digital competence] in the profession-oriented subjects.” (*prepared* Norway)

“[My] Masters in [integrating instructional technology in schools] prepared me for classroom instruction and design planning.” (*prepared* US)

Teachers also connected preparedness to their experience of using digital technologies in their personal lives such as, “I’ve used Skype privately, and recorded videos privately” (Norway). “Considering the fact that we live in a digital world, and use a computer/cell phone all day, the transition hasn’t been too bad” (Norway).

Participation in prior school-based digital learning initiatives (e.g., 1:1 and laptop computing) provided teachers the opportunity to experience digital tools and strategies in their classroom prior to the pandemic. *Prepared* teachers attributed a smooth transition to “ordinary” (Norway), “normal” (US) use of digital platforms and tools in the classroom. “Practice” (US and Norway) with digital tools over time (“I have been using elements of flipped learning and asynchronous learning for a few years.” US) was recognized as a part of online teaching readiness. Furthermore, schools that had turned to online learning to solve temporary school closings or other challenges prior to the pandemic, were ahead of the game when the pandemic hit. Teachers were able to practice online learning as a structured, and planned version of pandemic learning. “We were more prepared than most because we practiced Digital Learning to use instead of snow days…. When you practice, like a fire drill, it goes more smoothly when it’s the real thing” (US).

Similarly, being an online learner provided practice in an authentic setting and.

was connected to readiness as indicated by a *prepared* Norwegian teacher, “I have myself participated in online teaching at the [University], and also at the practice school.” Being an online learner brought insights about online learning that were useful when teachers shifted to the role of online teacher: “I had recently finished online studies at [University] in Norwegian and English and knew that this form of teaching requires much self-discipline” (*prepared* Norway). Finally, teachers, who themselves learned as online learners, were presented with models of online teaching from their graduate online instructors. As one *prepared* US teacher shared, “Due to taking grad classes online I was able to use that information from my teachers to guide me.”

The findings within the theme, *Trends in Preparation* the trends provide evidence that teachers’ preparedness for teaching online was linked to formal school-led training unrelated to the pandemic, emergency school-led training, and university education. School-led efforts were mainly focused on learning digital tools, aligning with the technical dimension of PEAT (DICTE, 2019). University education focused on theoretical, pedagogical, technical aspects. Only Norwegian teachers referred to the term *digital competence* in the elaborations.

All teachers who indicated they had completed a graduate program focused on online teaching, integration of technology in schools, or a course that focused on professional digital competence identified as *prepared* or *very well-prepared*. Given the evidence that teachers were “becoming more and more prepared,” addresses their ability to adapt technology use in their practice and reflects the attitudinal dimension of PEAT. School-led emergency efforts due to the pandemic potentially influenced teachers’ attitudinal dimension in a positive way. Referring to school-led emergency training, one *prepared* US teacher summarized, “I’ve been given the opportunity to really learn these new technology tools in a way that I probably wouldn’t have if I wasn’t thrown into it like we were. It will most likely result in my refining how I instruct next year.”

Teachers in this study who highlighted routine, applied uses of technology expressed a sense of preparedness. Experiences that situated tools in the context and culture of use played a pivotal role in preparedness. Alignment to PEAT (DICTE, 2019) dimensions primarily evident in the pedagogical dimension (e.g., flipped learning) and the technical dimension as many teachers representing this theme also mentioned the tools they used in their experiences. Furthermore, Norwegian teachers explicitly referred to *digital competence*. Although this is not a term typically used in US K12 education, US teachers did not explicitly use language from those standards and competency frameworks that inform digital learning in US schools and teacher education programs (i.e., ISTE Standards).

#### All about Tools

The theme *All about Tool*s characterizes the heavy focus of digital tools in teachers’ elaborations. Teachers reported a variety of digital tools that they used for presenting lessons, communications, and student assignment submissions within the first month of school closing. Table 3 presents a summary of tools specifically referenced by teachers in this study.

**Table 3:** Summary of digital tools referenced by teachers in the study

Tool Type	Norway	US
Synchronous Communication	Teams, WhatsApp, Skype, Messenger, Email	Teams, Google Meet, Zoom, Email
Learning Management Systems	OneNote Classroom, Google Classroom, Skooler, itslearning	Google Classroom, SeeSaw, Schoology, Nearpod
Apps and Internet	PowerPoint with voiceover and video recording, FlipGrid, Kikora, Screencast, OmniJoin, digital resources	Screencastify, FlipGrid, Voicethread, EdPuzzle, Narrated Powerpoints, other tools to make content videos, digital resources
Devices	iPads/tablets, Chromebooks/laptops, PC	iPads/tablets, Chromebooks/laptops

It is important to note that such a summary is not an exhaustive list of the technology used in both countries and the list; it is simply a representation of what respondents mentioned. It is likely that many more tools were available or used in schools.

The significance of this summary is that each tool represents the knowledge and skills needed to choose and use it appropriately for teaching. While the majority of *prepared* teachers reported prior experience using tools either for personal or professional use, both *prepared* and *unprepared* (Norway and US) teachers felt there was a “steep learning curve” (Norway). As one *prepared* Norwegian teacher summarized:

I feel like I was relatively prepared, the digital platforms were there and teachers and students knew them. But I did not realise until now the amount of possibilities that were actually available to use, and the learning curve has been steep - both to me and the students.

An *unprepared* Norwegian teacher also provided evidence of an increase in the number of tools presented to teachers and students at the onset of the pandemic and that these were not tools used prior to the crisis:

I have good digital skills but would love to have had more knowledge of more digital tools. It’s a lot to familiarize oneself within a short amount of time and it’s easy to choose ‘the first and best’ solution.

Access to digital technologies, devices and the internet was not a concern for teachers in Norway as both students and teachers had “access to the technology required for online teaching and learning” *(prepared* Norway). However access to devices and the internet was a major concern among US teachers as indicated in these statements:

“Chromebooks were available for all students to pick up. One problem is the lack of internet access in our rural county.” (*prepared* US)

“I am not prepared for how my students will access the material because they don’t have a laptop to learn synchronously or if they have internet.” (*unprepared* US)

Another finding related to tools was the perception that knowing the tools made the transition easy as evident in this representative quotation from a *prepared* teacher:

“We already use the internet anyway alongside in-person teaching, so it is relatively easy to continue only in digital mode.” (Norway)

Only a few teachers (3.0% Norway teachers; 3% US teachers) specifically distinguished between being *prepared* to use the tools and being *unprepared* to “teach online,” “differentiate learning,” or “use tools in an educational manner:”

“A bit prepared in the sense that we have used Teams for some time in the school. Not prepared for online TEACHING.” (Norway)

“I know how to use Google classroom, Microsoft teams, Khan academy and many other interfaces that teach kids online however this is usually integrated into the face-to-face classroom. I was not prepared on how to engage the kids or get them to care about learning online.” (US)

However, knowledge of how to use a tool seemed to define preparedness for the majority of teachers in this study:

“I can find resources online for students. I can create PowerPoints. I am able to use google and email. I have learned Zoom and Google Meet.” (*prepared* US)

“You are ready because you continue to use many of the same platforms as before the school shut down, it may be itslearning, e-mail and messenger /SMS. You are also ready with the way of thinking and the professional work is much the same before and after [the shutdown]: you prepare a theme, make a lesson plan and present it for your pupils.” (*prepared* Norway)

The theme, *All about Tools*, focused mainly on the technical dimension of PEAT (DICTE, 2019) in the sense that teachers, both *prepared* and *unprepared*, referred to their ability to use software and hardware and how these tools operate. Teachers in both countries recognized their technical ability, but also the lack of ability to teach with these tools within their content area as represented by the pedagogical dimension in PEAT. Teachers wrote about the need to learn new and different tools quickly with little time for practice as expected in an emergency situation. The lack of attention to the pedagogical dimension is understandable as pedagogical design and practices with technology was no longer situated in a familiar *professional practice*. The findings in this theme suggest a lack of education leader professional digital competence as “leadership in schools was far behind and was not prepared at all to take digital leadership.” (*prepared* Norway).

#### Agency without Autonomy

The theme *Agency without Autonomy* characterizes an interesting juxtaposition between teachers’ capacity for doing the work of teaching given the resources and limitations of the working environment, and perspectives on policies and actions by schools/districts/municipalities that prevented teachers from certain instructional actions or constrained instruction. Teachers’ statements representing their agency in finding “a way that can work in the various subjects” (*unprepared* Norway) included specific routes they took to learn tools such as “googling how to do certain things and learn[ing] from videos in YouTube” (*unprepared* US), learning “a lot along the way by testing various methods and hearing from my students what works and what doesn’t” (*unprepared* Norway), or simply, “jumping in with both feet!” Despite descriptions of “feeling overwhelmingly underprepared” and “helpless’’ from a few *unprepared* US teachers, there were more positive statements representing enthusiasm for learning new things in the midst of a difficult situation. Teachers expressed that “the move to online education has been painful but exciting” (*prepared* US). Even being unprepared did not deter a positive attitude in the transition to online teaching as exemplified by an *unprepared Norwegian* teacher:

To conduct online teaching has been an exciting way to teach. It’s challenged my capacity to adapt to new teaching situations and new tools, but I like to learn about new digital tools. I always think it’s exciting the things one can do with the help of technology.

Prepared Norwegian teachers, in particular, recognized “many positive aspects of distance education” such as, “students can control a lot of themselves, they become independent.” While not strongly represented in the data, it is still valuable to highlight that a few teachers in both Norway and the US elaborated that “trying new things - and it was so far very exciting (and can be useful later on)” (*prepared* Norway) had implications for the “future” (*prepared* US).

There were numerous references in both countries to the fact that “administrators are working together and helping us to implement new learning” (*prepared* US), however, teachers both *prepared* and *unprepared* emphasized challenges that did not honor or respect their professional knowledge, skills, and independence when it came to instructional decisions. Furthermore, the tool decisions made at the administrative level, lacked consideration for how these tools would be used by students and teachers, as exemplified by:

“We are relegated to using only programs that are approved by our school division whether they are intuitive and accessible or not.” (*unprepared* US)

“Higher ups continue to add paperwork to document attendance, contact, etc. in a number of ways rather than allowing us to just do what we do.” (*prepared* US)

“I’ve been doing this before, but I do not like having to be guided over to digital platforms with obvious weaknesses.” (*no indication* Norway)

“There’s no good platform provided by the government/school/municipality that we could use.” (*prepared* Norway)

The above statement shows that teachers lost their voice in the decision-making process as schools closed. Not to be deterred, teachers took the opportunity to exercise their professional independence and call for education reform:

“More youth report that they like this kind of school, so it can help to do that in the future, ‘a home-school day’ rather than go to school? (*prepared* Norway)

“I hope this crisis helps us to reevaluate what we are doing and starts to focus on the value of learning and knowledge rather than learning for a standardized test.” (*unprepared* US)

Similar to the theme, *All about Tools*, the findings in the theme, *Agency without Autonomy*, point to a lack of education leader professional digital competence. Support from school administrators was evident in teachers’ elaborations. However, as indicated in the theme, *Trends in Preparation*, the support was in the form of school-led training on tool use rather than pedagogical support. This theme provided evidence that school/district/municipality impeded teachers’ abilities to fully enact the pedagogical and technical dimensions. Yet, the constraints that limited their autonomy, teachers intentionally managed their professional growth and maintained a positive attitude toward the use of new technologies. The altitudinal dimension of PEAT (DICTE, 2019) was most evident in the theme, *Agency without Autonomy*. Despite challenges, many teachers from both Norway and the US took it upon themselves to learn the tools when adequate training was not available and adapt their teaching practice to what was considered a new learning environment for them and their students.

#### Influence of collaborations and networks

The theme, *Influence of Collaborations and Networks*, characterizes evidence of working, communicating, and collaborating with colleagues at the onset of school closings. This theme was more prevalent among Norway teachers with five times more references to collaboration and networks than in US teachers’ comments. Teachers enhanced their preparation for teaching online by working informally with other teachers. They indicated reliance on other teachers for ideas and supplementing formal education efforts. For example, the collaborations with colleagues played a role in one Norwegian teacher’ readiness in that “after some courses and colleague-guidance I feel quite ready to conduct online teaching. To date I have, and still am, conducting online teaching.” (*prepared* Norway). An unprepared US teacher was “not comfortable with new technology but…the support from [the] principal and colleagues [had] made all the difference.” A *well-prepared* Norwegian teacher “conducted meetings through Zoom, Skype, etc. with colleagues” before schools closed in order to practice with the tools. An *unprepared* Norwegian teacher had “learnt a lot thanks to our staff’s internal competence sharing and on Facebok [sic] groups for teachers and Youtube.”

Being remote proved “challenging to have good working relationships with colleagues.” (*prepared* Norway). But “daily meetings” (*prepared* Norway) provided “opportunities to test a lot of new, improved collaborations with colleagues” (*prepared* Norway). Colleagues were motivators, as one *unprepared* US teacher recalled, “During a whole-staff meeting recently, one colleague expressed excitement about this new challenge. He said, ‘This is what we trained for!’”.

The findings relating to collaboration/networking provide evidence that teachers’ preparedness for teaching online was also related to communication and networking. Teachers who had collaborated and conferred with colleagues on their digital practices and online teaching had positive attitudes to online teaching. This finding addresses the attitudinal dimension of PEAT (DICTE, 2019).

#### Challenges for work and learning lives

The theme *Challenges for Work and Learning Lives* characterizes teachers’ statements about working (teachers) and learning (students) in an online environment and the issue of privacy. The source of these comments came solely from the second data source of this study, the open-ended question on the survey that encouraged teachers to add anything else relevant to understanding their teaching practice during this crisis. Both Norwegian and US teachers frequently mentioned comments related to “working around the clock” (*prepared* Norway). One reason given for “working too much” and “not keep[ing] break times” (*prepared* Norway) was increased tasks and the “length of time to get a plan going” (*unprepared* US). For example, it was “difficult to spend much time on written feedback that would otherwise be given immediately face to face in the classroom” (unprepared Norway). As one teacher summarized, with online learning, “more time is required per lesson than in-person teaching” (*prepared* US). Other comparisons centered on interactions. One *prepared* US teacher best captured a sentiment that others mentioned, “Online is not the same and the social element that makes school fun for instructors and students has been taken away.”

Teachers shared concerns about the amount of time they and their students, particularly, young learners, were required to be on screen “for 12 hours a week” (unprepared US). Teachers were “tired of staring so much into a screen” (prepared Norway) and not only recognized but also experienced that “too much screen time causes headaches for teachers and students” (*unprepared* Norway.

However, both *unprepared* and *prepared* from both countries shared solutions to screen time issues such as, “Creating variety is extremely important in a period where there are many hours on the screen” (*unprepared* Norway) and “We are designing lessons that will encourage engagement, limit screen time and parental strain, and allow students to access the curriculum in nontraditional ways which foster creativity” (*prepared* US).

A prevalent topic presented by teachers was the notion of privacy. Privacy issues were discussed relative to teachers’ work-life balance as “the boundaries between work and private life [were] difficult to draw [with this] situation” (*prepared* Norway) as well as students’ school/life balance: “Navigating privacy of [Special Education] learners while trying to offer them online social interaction with other students has been difficult” (*very well-prepared* US).

Privacy was also mentioned in the context of digital technology access and use A prepared Norwegian

“Privacy has ironically made it extremely difficult to get access to the programmes at school.” (*prepared* Norway).

“When the platforms we use constantly crash, I create educational programs that may be available outside of school hours. It worries me a little that the privacy around me is not adequately safeguarded, as it now is image and sound files of myself online.” (prepared Norway).

“[It is] difficult to keep track of students’ schoolwork in the various subjects, while also safeguarding privacy considerations in various digital forums.” *(prepared* Norway).

This theme highlighted the ethical dimension of PEAT (DICTE, 2019) more than any other as there was evidence related to online responsibility and privacy. *Prepared* Norwegian teachers voiced a concern about a lack of “control” over students and their work. One prepared teacher mentioned that it was “more difficult to check that something is actually being done and that the students watch [the screen]. Furthermore, *prepared* Norwegian teachers were concerned about the “challenge to create a variety of assessment situations where students cannot cheat” and “fewer opportunities to prevent cheating.” As one prepared Norwegian teacher noted, “I see no possibility to randomly check to prevent cheating/collaboration.” Teachers in the US likely did not find this a concern as during the time frame that the TRIO survey was distributed US schools were focused on review and non-graded assignments.

In these findings, there are signs of pedagogical and attitudinal dimensions as teachers indicated they were creating a variety of ways to engage students to overcome challenges. However the ability to apply teachers’ existing digital competence to teaching online may not have been strong enough as teachers in both Norway and the US place more value on in-person learning as best represented by a *prepared* US teacher, who had completed a Master’s degree in e-Learning, “I definitely don’t want to be a virtual instructor.”

## Conclusion

Our conclusions summarize our main findings and lead to aspects necessary to address in the development of teacher education programs. Through the lens of digital competence, we examined primary and secondary Norwegian and US teachers’ perceptions and elaborations about their preparedness to teach online at the onset of pandemic-related school closing. Despite different standards and approaches to digital competence in the US and in Norway, our findings show similarities between Norwegian and US teachers’ sense of preparedness. It is evident that teachers’ preparation to teach online is to some extent, based on formal training or facilitated by school /district/municipalities. University teacher education that is focused on digital competence frameworks and guided by digital competence standards was shown to greatly influence teachers’ sense of preparedness to teach online. Teachers’ preparedness was also influenced by previous experiences as online learners, their personal experience with technology use, and collaboration with colleagues. Collaborations and networks, particularly for Norwegian teachers, contributed to positive attitudes regarding digital competence development.

In the US, there was a particular focus on students’ access to tools and the internet. In Norway, there was a concern for digital responsibility and online privacy issues. While concerns may have been different, teachers in both countries expressed enthusiasm to know more about the ways that online learning environments may better support students’ learning. Furthermore, teachers recognized the skills learned and practiced in online teaching and learning experiences expanded their repertoire for the future. This aligns with literature on transformative agency and the ways teachers “respond to challenges by transforming them into opportunities for their professional development” (Author, 2018, p. 1).

Teachers were also frustrated by a lack of support from education leaders on the one hand and lack of autonomy on the other. Support from education leaders was primarily related to the tools rather than pedagogical support for online teaching. At the same time teachers lost their voice in decision making processes as to which tools to apply. They also felt a loss of professionalism as education leaders made decisions that did not respect their experience as content experts, classroom teachers, and in some cases online learners, all prerequisites reported in the literature that support a transition to teaching online (Archambault & Crippen, 2009; Author, 2012) .

Elaborations on teachers’ preparedness to teach online through the lens of pedagogical, ethical, attitudinal, and technical dimensions of digital competence (DICTE, 2019) confirms a strong emphasis on technology access and use in the first weeks of the pandemic. The knowledge of how to use a tool seemed to define preparedness for the majority of the teachers. Standards related to digital competence have only recently shifted from focusing on digital tool competence to effectively integrating digital tools to provide instruction (Trust, 2017). Our findings show that barriers to online teaching in K12 settings first reported by Muilenburg and Berg (2003), particularly those related to *organizational resistance to change*, *lack of shared vision for distance education in organization*, *lack of strategic planning for distance education*, *lack of training provided, and slow pace of implementation*, remained impediments to online teaching at the onset of the pandemic. Therefore, it is not surprising that a decades-old focus on digital tools in schools still existed at the onset of the pandemic. Though first-order barriers had reportedly lessened (Durff & Carter, 2019) in the years prior to the pandemic, the lack of access to digital tools, suitable digital learning platforms and even the internet (US finding) were obstacles to learning as teaching transitioned to the online environment.

Other examples related to teachers’ digital competence relative to online teaching were highlighted through these PEAT dimensions. Some pedagogical strategies were shared such as the use of variety to deal with excessive screen time. Teachers identified issues related to privacy, online responsibility, and online wellness relative to the transition to online teaching. Acknowledgement of these aspects relate to the ethical dimension. The attitudinal dimension was most evident in relation to teachers’ agency. Despite challenges, many teachers from both Norway and the US took it upon themselves to learn the tools when adequate training was not available. Some worked to adapt technology use in their practice.

Decades of work have been done to develop teachers’ digital competence and more recently, support digital competence that includes online teaching. Digital competence frameworks (e.g., DICTE, 2019; Kelentrić et al., 2017), standards focused on integrating digital tools to provide instruction (e.g., Carpenter et al., 2020; International Society for Technology in Education, 2017), and standards for quality blended and online teaching (e.g., Powell et al., 2014; Quality Matters & Virtual Learning Leadership Alliance, 2019) were available to inform teachers and teacher education in the years prior to the rapid shift to online teaching as a result of the pandemic. Likewise barriers to effective digital technology use were first reported in the literature over 20 years ago (Ertmer, 1999; Muilenburg & Berg, 2003). We might expect from this literature that schools and teachers should have been relatively prepared to make a shift to online teaching. Yet, as this study indicated, there was a consequential number of teachers who were not prepared to transition to online teaching and even for those who indicated a sense of preparedness, essential elements of digital competence were missing.

The literature indicates that the definition of digital competence continues to evolve in Norway through policy-heavy frameworks for digital competence. The US has tried to resolve issues related to defining digital competence and quality online/blended learning through standards. Frameworks and standards often lack the concreteness that teachers need to apply in practice and therefore, their usefulness is uncommunicated to those who would most benefit. Not only did teachers lack a voice during the transition, their lack of voice in the development of digital competence policies, frameworks, and standards was uncovered in this study as indicated by statements both present and absent related to aspects of digital competence, particularly in the pedagogical and ethical dimensions. Without a voice, teachers are not challenged or encouraged to consider or define digital competence from their own perspectives and experiences. Future research should address ways to give teachers a bigger voice in the work to develop the concept of digital competence and opportunities to share their perspectives.

The significance of this study is that it provides a nuanced and dissected examination of the dimensions of digital competence to inform future efforts to develop teachers’ digital competence. The findings in this study warrant continued attention to the development of digital competence and what these findings mean across learning environments and for the different actors such as teachers, students, educational leaders, and authorities.

### Implications

Identifying trends from Norwegian and US teachers’ elaborations on their preparedness to teach online has led us to find interesting nuances between the countries which can be used to inform teacher educators on important steps in the future development of teachers’ digital competence as well as opportunities for teacher education programs in both countries.

1. There is a need to fully understand digital competence as multidimensional and with multifaceted pedagogical, ethical, attitudinal, and technical aspects as well as both knowledge and skills that transcend in-person teaching and learning experiences.
2. There is a need to fully understand online learning relative to digital competence. Online learning does not just include knowing the tools; rather it means attending to the ethical dimension, in ways that includes knowledge about privacy protections, online responsibilities, and digital health and wellbeing ness; it means attending to the attitudinal dimension, adapting to changes and technological developments, and being confident and resilient when trying out try new things.
3. There is a need to revisit and reframe barriers to effective technology use as applied across learning environments with updated digital competence frameworks and standards in mind. The literature on barriers, for the most part, were first conceived as online and blended learning were just emerging in primary and secondary education settings. The findings of this study highlight extrinsic first-order/intrinsic second-order (Durff & Carter, 2019; Ertmer, 1999; Tondeur et al., 2017), third-order (Tsai & Chai, 2012), and distance education (Muilenburg & Berg, 2003) barriers.
4. There is a need to model and support collaborations and networks. Teachers enhanced their preparation for teaching online by working informally with colleagues and established support networks on their own initiative. Additionally, early literature on barriers to the use of technology point to the collaboration as a way to address barriers. Teacher educators can model co-teaching environments in varied learning environments and provide opportunities for collaboration not only on project work, but in processes of finding solutions to problems of practice.
5. Furthermore, teacher education programs need to focus on varied teaching modes and not only either in-person teaching or online teaching. Student and inservice teachers need to get acquainted to teach on different platforms (online/offline) and in blended environments. Teachers’ professional digital competence should also include knowledge and training in various blended learning approaches.
6. There is a need to address the development of education leaders’ professional digital competence relative to all learning environments and delivery modalities. Digital competence does not only apply to teachers and students, but school leaders as well (International Society for Technology in Education, 2018).
7. There is a need to model and support collaborations and networks. Teachers enhanced their preparation for teaching online by working informally with colleagues and established support networks on their own initiative. Additionally, early literature on barriers to the use of technology point to the collaboration as a way to address barriers. Teacher educators can model co-teaching environments in varied learning environments and provide opportunities for collaboration not only on project work, but in processes of finding solutions to problems of practice.

Findings and implications from this study show that the pandemic provided an opportunity for reexamining standards and competences focused on digital learning in ways that influence teaching practice. Situating this study in the literature, standards, conceptualizations, and definitions that influence teacher education in both Norway and the US exposed us to new perspectives we see as important to address in future teacher education. Drawing from different perspectives on shared concepts not only leads to richer practice but also identifies potential solutions to problems of practice that might not have been considered and can therefore serve as excellent topics for future research.

## Data Availability

The datasets generated during and/or analyzed during the current study are available from the corresponding author on reasonable request. The survey itself is available her in all the languages: https://www.uv.uio.no/ils/english/research/projects/trio/.
